# Dietary Quality Associated with Self-Reported Diabetes, Osteoarthritis, and Rheumatoid Arthritis among Younger and Older US Adults: A Cross-Sectional Study Using NHANES 2011–2016

**DOI:** 10.3390/nu13020545

**Published:** 2021-02-07

**Authors:** Masako Matsunaga, Eunjung Lim, James Davis, John J. Chen

**Affiliations:** Department of Quantitative Health Sciences, John A. Burns School of Medicine, University of Hawaii at Manoa, Honolulu, HI 96813, USA; jamesdav@hawaii.edu (J.D.); jjchen@hawaii.edu (J.J.C.)

**Keywords:** dietary patterns, dietary quality, healthy eating index, diabetes, arthritis, osteoarthritis, rheumatoid arthritis

## Abstract

Background: To date, few studies have compared the dietary quality of US adults with diabetes mellitus (DM), osteoarthritis (OA), and rheumatoid arthritis (RA) by age groups. Methods: This study used cross-sectional data from adult participants from National Health and Nutrition Examination Survey 2011–2016 to identify dietary quality measured by Healthy Eating Index (HEI)-2015 total and component scores and self-reported disease status for DM, OA, and RA. Associations between the disease status and HEI-2015 total/component scores among younger adults aged 20–59 years (*n* = 7988) and older adults aged 60 years and older (*n* = 3780) were examined using logistic regression models. These accounted for the complex survey design and were adjusted for self-reported disease status, sex, race/ethnicity, education levels, income status, weight status, physical activity levels, and smoking status. Results: Among younger adults, 7% had DM, 7% had OA, and 3% had RA. Among older adults, 20% had DM, 32% had OA, and 6% had RA. Moderate added sugar intake was associated with diabetes in all adults. Excess sodium intake was associated with DM among younger adults. Inadequate seafood and plant protein intake was associated with RA among younger adults, while a poor overall dietary pattern was associated with RA among older adults. Conclusions: The dietary quality of US adults varied by self-reported DM, OA, and RA status, and each varied by age group.

## 1. Introduction

Diabetes mellitus (DM) and arthritis (AR) are common chronic diseases and among the major contributors to the growing burden of healthcare costs in the United States [1,2,3]. The estimated direct medical costs and earning losses total more than $300 billion for DM and AR [1,3]. These diseases are also among the leading causes of disabilities. The prevalence rates of DM and AR in those with any disability are more than doubled compared to those without disability [4]. Furthermore, previous studies reported a high prevalence of AR among individuals with DM and vice versa [5,6,7,8,9,10], which has motivated researchers to examine the links between the two diseases [11,12,13,14,15].

Osteoarthritis (OA), the most common type of AR [10], is characterized by a loss of cartilage along with bone hyperplasia, sclerosis of subchondral bone, and synovial membrane inflammation at the joints [11]. Older age, obesity, family history, and mechanical stress on the joints are known risk factors for OA [5,6,11,16]. In addition, recent studies have discussed metabolic disturbances as a phenotype of OA [5,11,12,16]. Rheumatoid arthritis (RA) is the most common type of autoimmune AR. The prominent feature of this disease is symmetrical pain and swelling of the hand, wrist, and knee joints, but other joints could be affected [17]. Older age, female, family history, and tobacco smoking were considered risk factors in past studies [7,18,19]. As a result of the high prevalence of DM among patients with AR, studies have been conducted on the mechanism of systematic inflammation involved in these diseases [7,14,15,20]. However, past studies have not focused on the dietary link between DM and AR, such as common dietary patterns among those patients.

Developing healthy dietary recommendations for people at higher risk for common chronic diseases can be one strategy to help them prevent and delay their onset. Numerous studies have used dietary quality indexes, such as the Healthy Eating Index (HEI) [21,22,23,24,25,26] to examine associations between dietary quality and chronic diseases. For example, a meta-analysis study reported that the highest quality diet, as assessed by dietary indexes, resulted in a significant risk reduction for type 2 DM [26]. For adults diagnosed with type 2 DM, a variety of healthy dietary patterns can be individualized to meet their personal lifestyle management goals [27,28]. On the other hand, most past nutrition studies for AR focused on the Mediterranean dietary pattern [29,30,31,32,33,34]. Although improvements in symptoms for OA and RA patients eating the Mediterranean diet have been documented, its effect on prevention appears to be still inconclusive [29,30]. Moreover, this dietary pattern may not be a practical choice for all patients with AR due to differences in food culture and availability.

HEI-2015 is a measure for assessing dietary quality [22], specifically the degree to which a set of foods aligns with the Dietary Guidelines for Americans (DGA) 2015–2020 [35]. The DGA covers various dietary patterns that reflect cultural, ethnic, traditional, and personal preferences and tolerances, as well as food costs and availability [22]. Thus, the dietary components assessed by this index provide relevant and practical information to most US adults. As older adults have higher risks for chronic diseases and healthier food patterns [36], the association between dietary quality and chronic diseases should be assessed separately for different age groups. However, analysis stratified by age group is not feasible unless a large dataset is available. To the best of the authors’ knowledge, no study compared associations between dietary quality and common chronic diseases: DM, OA, and RA, stratified by age group, using nationally representative data. The purpose of this study is to examine the associations between the HEI-2015 total and component scores and self-reported DM, OA, and RA, using the National Health and Nutrition Examination Survey (NHANES) data.

## 2. Materials and Methods

### 2.1. Data and Study Participants

This study used data from adult participants in three NHANES cycles (2011–2012, 2013–2014, 2015–2016) [37]. NHANES is a cross-sectional population-based survey utilizing a complex, multistage probability cluster design to select a sample representative of the civilian non-institutionalized US resident population. NHANES 2011–2016 procedure and protocols were reviewed and approved by the National Center Health Statistics Research Ethics Review Board. NHANES obtained written, informed consent for all adult participants. Detailed information on NHANES is available elsewhere, and the data are publicly available on the NHANES website (https://www.cdc.gov/nchs/nhanes, accessed on 15 September 2020). In the three cycles, a total of 29,902 individuals provided information to NHANES. In this study, participants aged 19 years or younger (12,854 individuals) were excluded from the study sample. After excluding those who did not provide two reliable 24-h dietary recalls (4056 individuals) and information on important variables considered for the multivariable regression models (1224 individuals), 11,768 study participants were included in the analysis.

### 2.2. Self-Reported Disease Status

Diabetes mellitus (DM) status was determined by responses to the Diabetes questionnaire. Participants were asked if s/he had been ever told by a doctor or health professional that s/he had diabetes or sugar diabetes other than during pregnancy. Those who answered “yes” were defined as with DM. Those who answered “no” or “borderline” were defined as without DM. Arthritis (AR) status was determined by responses to the Medical Condition questionnaire. Those who answered that a doctor or health professional had not told s/he had arthritis were defined as without AR. Those who self-reported “osteoarthritis” were defined as with OA, and those who self-reported “rheumatoid arthritis” were defined as with RA. Those who self-reported “other arthritis” were defined as with other AR.

### 2.3. Healthy Eating Index-2015

The HEI-2015 consists of nine adequacy components (Total Fruits, Whole Fruits, Total Vegetables, Greens and Beans, Whole Grains, Dairy, Total Protein Foods, Seafood and Plant Proteins, and Fatty Acids) and four moderation components (Refined Grains, Sodium, Added Sugars, and Saturated Fats) [22,23]. Total Fruits, Whole Fruits, Total Vegetables, Greens and Beans, Total Protein Foods, and Seafood and Plant Proteins are assigned five points each. The rest of the components are assigned 10 points each. Higher scores indicate higher intakes for adequacy components, and maximum scores indicate a person’s intake reaches the recommended level. Higher scores indicate lower intakes for moderation components, and maximum scores show a person’s intake is equal to or lower than the recommended level. A total score is calculated by adding up the 13 component scores, a maximum score of 100; a higher score indicates a better overall dietary quality. In this study, scores were calculated for each participant from their two 24-h dietary recalls using the simple HEI scoring algorithm method (per person) [38]. The HEI total and component scores were categorized using the cut-points based on the overall sample’s score distribution. The HEI total score was categorized into quintiles (Q1–Q5), with Q1 including those with the lowest scores. The HEI-2015 components were categorized in tertiles (Q1–Q3: low, medium, or high). However, Greens and Beans, Total Protein Foods, and Seafood and Plant Proteins were highly skewed with narrow ranges of scores (0–5 points). Since we could not find cutoff points for tertiles making approximately 1/3 each, we dichotomized these components using the median (Q1–Q2: low or high).

### 2.4. Sociodemographic Characteristics

Metabolic equivalents minutes per week (MET-minutes) were calculated for each participant based on responses to the Physical Activity questionnaire. Using the calculated MET-minutes, participants were classified into three physical activity levels (low: <150, medium: 150–<300, or high: ≥300 MET-minutes), which were defined by the global recommendations for physical activity for health [39]. Income status was classified into three levels of the poverty income ratio (PIR) (low: <130, medium: 130–<350, or high: ≥350%), which indicates a ratio of family income to the US poverty guidelines. These cutoff points were used in previous studies using NHANES data [40,41], and the PIR of 130% is the income eligibility criterion for the Supplemental Nutrition Assistance Program [42]. Other sociodemographic characteristics included were sex (male or female), race/ethnicity (Non-Hispanic white, Non-Hispanic black, Mexican American, other Hispanic, Non-Hispanic Asian, or other race), weight status (underweight/healthy: body mass index (BMI) < 25, overweight: 25 ≤ BMI < 30, or obese: BMI ≥ 30 kg/m^2^), education levels (less than high school graduate, high school graduate, some college, or college graduate or above), and smoking status (current smoker or non-current smoker).

### 2.5. Statistical Analysis

All analyses were performed to account for the complex sample survey design of NHANES with primary sampling units, strata, and dietary two-day sample weights (WTDR2D). Variances were estimated by the Taylor series linearization method [43,44]. Analyses were stratified by two age groups—younger adults (20–59 years; *n* = 7988) and older adults (60 years or older; *n* = 3780). Weighted percentages of sociodemographic and dietary characteristics (HEI-2015 total and component scores) were obtained for each age group by disease status. The Rao-Scott adjusted chi-squared tests were performed to assess the independence of DM status. Chi-squared tests with Monte Carlo simulations based on weighted percentages of characteristics were used to assess the independence of AR status. Furthermore, post-hoc pairwise chi-squared tests with Bonferroni adjustments were performed for significant variables to assess the difference between two groups within AR status (without AR vs. with OA, without AR vs. with RA, with OA vs. with RA). Multivariable logistic regression analyses were conducted to examine associations between dietary quality and each disease status. The outcome was yes or no for DM, OA, or RA. Initial models included risk factors for DM or AR (self-reported disease status, sex, weight status, physical activity level, and smoking status) [17,18,19,45] and factors that may affect an individual’s dietary pattern (race/ethnicity, education level, and income status). Final models were determined by a backward stepwise-selection process. Odds ratios (ORs) and their 99% confidence intervals (CIs) were estimated from the results of the initial and final logistic regression models. Sensitivity analyses were performed after excluding participants with extremely high or low energy intakes (<1st percentile and >99th percentile). A *p*-value of less than 0.01 was considered statistically significant due to multiple testing issues. Statistical analyses were conducted using the survey package in R version 4.0.2. [46].

## 3. Results

### 3.1. Sociodemographic Characteristics of Younger and Older US Adults by Self-Reported Disease Status

Table 1 shows the sociodemographic characteristics of younger US adults by disease status. Among younger adults, 7% had DM, 7% had OA, and 3% had RA. Compared with younger adults without DM, younger adults with DM reported a higher proportion of AR, education levels less than college graduate, obesity, and low physical activity level. Compared with younger adults without AR, a greater proportion of younger adults with OA had DM. They were also more likely to be female, non-Hispanic white, current smoking, and more likely to have some college education or less, obesity, and low physical activity level. Compared with younger adults without AR, younger adults with RA had similar characteristics to younger adults with OA. Additionally, a greater proportion of younger adults with RA was non-Hispanic blacks and had low income.

Table 2 shows the sociodemographic characteristics of older US adults by disease status. Among older adults, 20% had DM, 32% had OA, and 6% had RA. Older adults with DM had the same characteristics as younger adults with DM for weight status and physical activity levels. In addition, older adults with DM were more likely to be male, not non-Hispanic white, and they were more likely to have an education level of high school graduate or lower and low or medium income. Compared with older adults without AR, older adults with OA had similar characteristics to younger adults with OA. A greater proportion of older adults with OA was found in the categories for DM, female, non-Hispanic white, obesity, and low physical activity level. Older adults with RA had the same characteristics as younger adults with RA. They were more likely to be with DM, female, non-Hispanic black, and more likely to have education levels below college graduate, low income, obesity, and low physical activity level.

### 3.2. Dietary Characteristics of Younger and Older US Adults by Self-Reported Disease Status

Table 3 presents the dietary characteristics of younger US adults by disease status. No significant difference was found in the HEI-2015 total score between the two DM status groups and across the three AR status groups. However, some variations were found in the HEI-2015 components scores. A greater proportion of younger adults with DM appeared to have a score categorized in Q1 for Sodium (44%) and Saturated Fats (44%) and in Q3 for Added Sugars (45%). No significant difference was found between younger adults without AR and with OA in all the HEI-2015 components. A greater proportion of younger adults with RA appeared to have a score categorized in Q1 for Greens and Beans (62%), Seafood and Plant Proteins (68%), and Added Sugars (44%) than younger adults without AR.

Table 4 presents the dietary characteristics of older US adults by disease status. A significant difference in the HEI-2015 total score was not found between the two diabetes status groups and between the groups without AR and with OA. In contrast, a greater proportion of those with RA had a score categorized in Q1 of the HEI-2015 total score (without AR: 11%; with OA: 12%, with RA: 22%). The HEI-2015 component scores of older US adults also appeared to vary by disease status. A greater proportion of older adults with DM appeared to have a score categorized in Q3 for Total Protein Foods (76%), Q1 for Sodium (40%), and Q3 for Added Sugars (46%), compared with older adults without DM. A greater proportion of older adults with OA appeared to have a score categorized in Q2 for Total Fruits (37%) and Whole Fruits (38%), Q3 for Sodium (41%), and Q2 for Added Sugars (41%) and Saturated Fats (36%), compared with older adults without AR. A greater proportion of those with RA appeared to have a score categorized in Q1 for Total Fruits (27%) and Greens and Beans (62%), compared with those without AR. Compared with older adults with OA, fewer older adults with RA were categorized in Q3 for Whole Fruits (32%).

### 3.3. Odds Ratios of Having Self-Reported Diabetes Mellitus, Osteoarthritis, or Rheumatoid Arthritis among Younger and Older US Adults

Table 5 shows the ORs and 99% CIs from the initial multivariable logistic regression models for HEI-2015 total scores. After adjusting for potential confounders, an HEI-2015 total score was not associated with the self-reported diseases in both age groups except for RA in older adults. The results show that older adults with a total score categorized in Q4 were 65% less likely to have RA than older adults with a total score categorized in Q1 (OR = 0.35, 99% CI 0.16–0.76). Even after removing insignificant covariates, an HEI-2015 total score was not significant for DM and OA in both age groups and RA in younger adults. On the other hand, the final model for RA in older adults shows a similar OR for Q4 vs. Q1 (OR = 0.39, 99% CI 0.18–0.82).

The results of the initial multivariable logistic regression analysis for the HEI-2015 components show that most food group and nutrient intakes were not associated with the self-reported disease (Table 5). However, younger adults in Q3 for Whole Grains were more likely to have DM than younger adults in Q1. Meanwhile, younger adults in Q3 for Saturated Fats were less likely to have DM than younger adults in Q1. In all adults, those in Q3 for Added Sugars were more likely to have DM than those in Q1. The final model results (Table 6) show that younger adults in Q3 for Sodium were 44% less likely to have DM than younger adults in Q1 (OR= 0.56, 99% CI 0.33–0.95). Younger adults in Q3 for Added Sugars were 2.31 times more likely to have DM than younger adults in Q1 (99% CI 1.33–4.02). Meanwhile, the odds for younger adults in Q3 for Whole Grains and Saturated Fats became less contrasting to the odds for younger adults in Q1 for the component, after excluding insignificant variables. Added Sugars remained in the final model for older adults, suggesting that older adults in Q3 were 2.54 times more likely to have DM than older adults in Q1 (99% CI 1.62–3.97).

No significant HEI components were found in the initial model results for OA in younger and older adults (Table 5). The final model results for younger adults continued to show no significant HEI-2015 component. However, the final model for older adults found that those in Q2 (medium level) for Saturated Fats were 1.54 times (99% CI 1.08–2.19) likely to have OA than those in Q1 (Table 6).

The initial models for RA in younger adults found no significant HEI-2015 components. However, the initial model in older adults found that those in Q3 for Whole Fruits were less likely to have RA than those in Q1 (Table 5). The final model results (Table 6) found that younger adults in Q2 for Seafood and Plant Proteins were 42% less likely to have RA than younger adults in Q1 (OR = 0.58, 99% CI 0.35–0.92). Older adults with a score categorized in Q3 for Whole Fruits were 55% less likely to have RA than those in Q1 (OR = 0.45, 99% CI 0.22–0.92) when Total Fruits was included in the model. However, after removing Total Fruits, Whole Fruits no longer had a significant OR (0.62, 99% CI 0.33–1.19).

The final results (Table 6) also show obese adults (BMI ≥30 kg/m^2^) had a higher odds of DM and OA than those with a BMI of less than 25 kg/m^2^. Those who graduated college had lower odds of DM and RA than those who did not graduate high school. Younger adults with OA or RA had higher odds of DM, and younger adults with DM had higher odds of OA or RA. However, this pattern was not found in older adults. Sensitivity analyses were performed after those who had less than the 1st percentile (688 kcal) or more than the 99th percentile (4444 kcal) of averaged energy intake per day were excluded. The results showed similar results (results not shown).

## 4. Discussion

This cross-sectional study examined US adults’ dietary quality by self-reported DM, OA, and RA status using HEI-2015 scores calculated from 2-day dietary recall data collected from adult participants in NHANES 2011–2017. Since age is a significant factor for dietary quality and risks for these chronic diseases, we investigated the dietary quality among younger and older adults separately. Our investigation may be the first study to assess dietary quality association with DM, OA, and RA in US adults by age group using data from a nationally representative sample. Our results show no association between overall dietary quality and self-reported diseases, except for RA in older adults.

Regarding the HEI-2015 components, a few dietary components were associated with DM. In contrast, one or none of the HEI-2015 components was associated with OA and RA, with variations between younger and older adults. These findings suggest that varying dietary quality exists across disease and age groups. The proportion of those with DM that would be categorized in the top quantile of Added Sugars was higher in both age groups, and Added Sugars remained in the final logistic regression models for both age groups. This finding implies that adults with DM may be more compliant with the recommendation for added sugar intakes regardless of age differences. However, our results also show unhealthy dietary patterns for sodium intake in both age groups of adults with DM and saturated fat intakes in younger adults. Sodium intake was still associated with DM in younger adults after adjusting for potential cofounders. These findings suggest that consuming appropriate amounts of sodium and saturated fats appears to be challenging for a high proportion of adults with DM. A past study that examined dietary quality among US adults with and without DM using the HEI-2010 found a lower mean score for sodium intake among adults with DM [47]. Since the current study assessed dietary quality using the HEI-2015, which includes Saturated Fats, we also found their suboptimal saturated fat intake. Therefore, to help adults with DM improve their overall dietary quality, moderate intakes of foods high in sodium and saturated fats may need to be emphasized along with healthy dietary patterns.

The current study did not find significant differences in the HEI-2015 components between younger adults without AR and with OA. Older adults with OA showed the likelihood of intakes of fruits in any form, added sugars, and saturated fats, which would be categorized as the average level (Q2), were higher than among older adults without AR. Still, any dietary component’s lowest score level was not associated with OA after adjusting for potential confounders. Meanwhile, the dietary quality of US adults with RA appears to differ from adults without AR and adults with OA. First, suboptimal dietary patterns for seafood and plant proteins were detected in younger adults with RA. Seafood and Plant Proteins remained in the final logistic regression model, suggesting a lower odds of RA in those in Q2 compared with those in Q1. Past studies reported that the Mediterranean dietary pattern, fish consumption, and fish oil supplementation helped reduce pain and increase physical function in people living with RA [30,33,48,49]. However, there remains uncertainty about the effects of these nutritional interventions for preventing RA [30,33]. Meanwhile, the benefits from adequate intake of plant proteins have not been well-studied. Inadequate intakes of seafood and or plant proteins could be related to the mechanism of RA pathogenesis, as the association was observed only in younger adults. Nevertheless, increasing intakes of this food group may help younger adults with RA improve their dietary quality and manage their RA. Further interventions are required to evaluate the relationship between seafood and plant proteins intake and RA.

Our findings suggest decreasing odds of having RA for older adults with a high HEI-2015 total score than older adults with a low HEI-2015 total score. The results regarding dietary characteristics suggest that older adults with RA were more likely to have inadequate fruit intakes, which could be related to their health conditions and sociodemographic characteristics. The primary treatment for RA relies on medications, such as disease-modifying antirheumatic drugs (DMARD), to manage pain and inflammation and minimize additional joint damage [17]. Patients with long-standing and inadequately treated RA could develop severe joint damage and deformities of the hands [17]. Thus, practicing a healthful dietary pattern could be challenging for some older patients [50,51]. Since older adults with a low-education or low-income level had lower rates of DMARD receipt [52,53], dietary recommendations for patients with RA should be individualized considering their current medications, RA activities, and sociodemographic status [54]. Unfortunately, nutrition counseling for patients with RA is not always considered an essential component of comprehensive health care. Unlike nutrition counseling for DM, it is not covered by Medicare insurance [55]. However, it could provide benefits to patients with RA and potentially reduce total medical costs. For example, nutrition counseling would be an excellent opportunity to introduce science-based healthful dietary patterns, such as the HEI-2015. It would also be an opportunity to assess if the patient has been following any inappropriate elimination diet [31] or a fad diet [56]. As frequent food allergies and tolerances in those with RA have been documented [57], nutrition counseling would also allow patients to learn about alternative foods. These could help them avoid nutrient deficiency by eliminating foods causing allergic or physical unpleasant reactions.

The results showed that the proportion of adults with a BMI categorized as obesity (≥30 kg/m^2^) was greater among those with any self-reported diseases than their counterparts. Obesity remained significant in the final logistic regression models for DM and OA in both age groups. Thus, balanced energy intake and gradual reduction of excess weight are essential topics in dietary education for all adults, especially adults with DM and or OA. Adults with a low-education and or low-income level are more likely to lack knowledge of typical daily calorie requirements [58]. In this regard, additional assistance may be needed for those individuals. For older adults in general, guidance toward appropriate energy intake with nutrient-dense foods is essential to help them avoid muscle loss and maintain physical function. Since higher risks for complications and poor prognosis in underweight patients, such as the high risk of cardiovascular diseases in those with rheumatoid cachexia, has been well documented [59,60], additional assistance is also necessary for underweight older adult patients. Therefore, nutritional counseling may help all older adults, especially those with DM and or RA, improve their dietary quality and manage their chronic diseases.

Limitations of this study include its cross-sectional design and reliance on self-reported dietary data and disease status. Using categorical variables of HEI-2015 total and component scores in the logistic regression analysis may result in inadequate control of confounding. Subtypes of DM were not considered in the analysis; thus, the results may not be generalizable to all subtypes of DM. In addition, many participants reported they had AR but did not know what type. Since OA is the most common form [8,16], many of them possibly had OA. As a result, the findings for OA may not fully reflect the true dietary characteristics of adults with OA. Although examining dietary characteristics of adults who have both DM and AR (either of OA or RA) could provide more insights into the links between the two diseases, analyses were not conducted for this categorization due to inadequate sample size. Further research is required to elucidate the dietary quality of adults with DM and OA and those with DM and RA. Lastly, our results may not be generalized to those living in the region with different food availability and living environments, e.g., those in developing countries.

Despite these limitations, the study has several important strengths. The analysis was conducted separately for younger and older adults using three cycles of NHANES. Since this study used the recent data that were collected from national representatives through well-designed protocols, the results reflect general US adult dietary quality by self-reported DM and AR status and age groups and the associations between dietary quality and common chronic diseases. Furthermore, these results were based on the HEI-2015 scores calculated from 2-day dietary recalls, which could produce less biased estimates for dietary quality than estimates based on 1-day dietary recall data [61]. This study also provides information on the magnitude of the associations of dietary risk factors and other sociodemographic characteristics, which could help develop effective and individualized dietary recommendations by taking account of an individual’s sociodemographic characteristics. Finally, the results suggest that many US adults still need to improve their dietary patterns, as the majority appeared to receive a D (60–69 points) or F (0–59 points) in the HEI-2015 total score based on the grading system suggested by the HEI-2015 developer [22]. Thus, achieving the perfect-score level for each of the HEI components would be the final goal for all US adults to practice healthful dietary patterns suggested by the DGA [35].

## 5. Conclusions

This study shows varying dietary quality and sociodemographic characteristics along with disease status and age groups, and the varying associations between dietary quality and self-reported diseases in each age group. These results suggest that depending on a patient’s health condition, age, and sociodemographic characteristics, different dietary components may need to be addressed in nutrition counseling. The findings may help guide the development of a nutritional screening plan, create an assessment tool to improve the dietary quality of adult patients with these diseases, and formulate research plans to study links between DM and AR.

## Figures and Tables

**Table 1:** Sociodemographic characteristics of younger US adults by self-reported diabetes mellitus status and arthritis status: National Health and Nutrition Examination Survey (NHANES) 2011–2016.

	All Younger Adults *n* = 7988	Without DM *n* = 7361 (93.5%)	With DM *n* = 627 (6.5%)	Without AR *n* = 6743 (83.6%)	With OA *n* = 496 (7.0%)	With RA *n* = 256 (2.8%)
Self-Reported AR						
Without AR	6743 (83.6)	6323 (85.1)	420 (62.4)			
With OA	496 (7.0)	415 (6.4)	81 (15.4)			
With RA	256 (2.8)	209 (2.5)	47 (7.5)			
Other AR	493 (6.7)	414 (6.1)	79 (14.8)			
*p*-value			<0.001 *			
Self-Reported DM						
Without DM	7361 (93.5)			6323 (95.1)	415 (85.6)	209 (82.4)
With DM	627 (6.5)			420 (4.9)	81 (14.4)	47 (17.6)
*p*-value				<0.001 *		<0.001 *
Sex						
Male	3742 (48.8)	3451 (48.9)	291 (46.9)	3289 (51.1)	181 (34.9)	91 (36.6)
Female	4246 (51.2)	3910 (51.1)	336 (53.1)	3454 (48.9)	315 (65.1)	165 (63.4)
*p*-value			0.487	<0.001*		<0.001*
Race/Ethnicity						
Non-Hispanic White	3019 (62.5)	2827 (63.1)	192 (54.2)	2395 (60.6)	280 (76.8)	109 (66.0)
Non-Hispanic Black	1786 (11.8)	1609 (11.4)	177 (16.5)	1487 (11.8)	104 (10.2)	69 (14.7)
Mexican American	1076 (10.0)	967 (9.8)	109 (12.0)	973 (10.9)	26 (3.7)	33 (7.5)
Other Hispanic	786 (6.4)	718 (6.3)	68 (7.9)	682 (6.8)	38 (3.5)	29 (8.3)
Non-Hispanic Asian	1010 (5.9)	953 (6.0)	57 (5.0)	956 (6.7)	18 (1.8)	5 (0.9)
Other race	311 (3.4)	287 (3.3)	24 (4.5)	250 (3.2)	30 (3.9)	11 (2.5)
*p*-value			0.018	0.003 *		0.003 *
Education Levels						
<High school	1363 (12.7)	1205 (12.3)	158 (18.4)	1120 (12.3)	79 (13.6)	68 (20.5)
High school graduate	1692 (20.5)	1553 (20.3)	139 (22.1)	1393 (20.1)	108 (18.7)	54 (22.8)
Some college	2585 (33.4)	2377 (33.0)	208 (39.6)	2147 (32.6)	184 (40.4)	90 (39.9)
≥College graduate	2348 (33.4)	2226 (34.3)	122 (19.8)	2083 (35.0)	125 (27.4)	44 (16.9)
*p*-value			<0.001 *	0.009 *		<0.001 *
Income Status (PIR)						
Low (<130%)	2667 (24.7)	2417 (24.3)	250 (30.7)	2171 (24.2)	176 (24.6)	122 (36.5)
Medium (130–350%)	2787 (33.4)	2567 (33.2)	220 (36.0)	2391 (33.6)	152 (30.6)	74 (36.5)
High (≥350%)	2534 (42.0)	2377 (42.6)	157 (33.3)	2181 (42.2)	168 (44.8)	60 (27.0)
*p*-value			0.035		<0.001 *	<0.001 *
Weight Status						
Underweight/Healthy	2438 (31.0)	2371 (32.4)	67 (10.0)	2203 (33.0)	98 (22.3)	39 (24.8)
Overweight	2419 (32.0)	2273 (32.9)	146 (19.1)	2106 (33.1)	122 (25.4)	75 (24.1)
Obese	3131 (37.0)	2717 (34.6)	414 (70.9)	2434 (34.0)	276 (52.3)	142 (51.1)
*p*-value			<0.001 *	<0.001 *		<0.001 *
Physical Activity Levels ^a^						
Low	2639 (30.1)	2355 (29.0)	284 (45.9)	2094 (27.5)	220 (44.5)	108 (40.7)
Medium	847 (10.3)	790 (10.5)	57 (6.6)	731 (10.3)	42 (7.6)	20 (12.2)
High	4502 (59.6)	4216 (60.4)	286 (47.5)	3918 (62.2)	234 (48.0)	128 (47.1)
*p*-value			<0.001 *	<0.001 *		<0.001 *
Smoking Status						
Non-current smoker	6230 (78.4)	5734 (78.3)	496 (79.6)	5390 (80.5)	330 (68.1)	168 (63.3)
Current smoker	1758 (21.6)	1627 (21.7)	131 (20.4)	1353 (19.5)	166 (31.9)	88 (36.7)
*p*-value			0.677	<0.001 *		<0.001 *

Abbreviations: NHANES National Health and Nutrition Examination Survey; DM diabetes mellitus; AR arthritis; OA osteoarthritis; RA rheumatoid arthritis; PIR poverty income ratio. Numbers presented are unweighted counts and weighted percentages based on the NHANES complex survey design. *P*-values for without vs. with DM (in the with DM column) were obtained by Rao-Scott adjusted chi-square tests. *P*-values for without AR vs. with OA (in the without AR column), with OA vs. with RA (in the with OA column), and without AR vs. with RA (in the with RA column) were obtained by Bonferroni adjusted post-hoc pairwise chi-square tests, using Monte Carlo simulations based on the weighted percentages. Analysis by AR status included those with other AR: characteristics of those with other AR are not shown in tables. ^a^ Low = <150, medium = 150–<300, and high = ≥300 metabolic equivalents minutes per week. *—indicates a statistically significant difference with a *p*-value of less than 0.01.

**Table 2:** Sociodemographic characteristics of older US adults by self-reported diabetes mellitus status and arthritis status: NHANES 2011–2016.

	All Older Adults *n* = 3780	Without DM *n* = 2828 (80.0%)	With DM *n* = 952 (20.0%)	Without AR *n* = 1864 (46.6%)	With OA *n* = 933 (31.5%)	With RA *n* = 314 (5.8%)
Self-Reported AR						
Without AR	1864 (46,4)	1448 (48.0)	416 (40.1)			
With OA	933 (31.5)	685 (30.9)	248 (33.9)			
With RA	314 (5.8)	215 (5.3)	99 (7.9)			
Other AR	669 (16.3)	480 (15.8)	189 (18.0)			
*p*-value			0.041			
Self-Reported DM						
Without DM	2828 (80.0)			1448 (82.7)	685 (78.5)	215 (72.8)
With DM	952 (20.0)			416 (17.3)	248 (21.5)	99 (27.2)
*p*-value				<0.001 *		<0.001 *
Sex						
Male	1845 (46.9)	1332 (44.6)	513 (56.0)	1075 (54.8)	326 (33.8)	134 (40.9)
Female	1935 (53.1)	1496 (55.4)	439 (44.0)	789 (45.2)	607 (66.2)	180 (59.1)
*p*-value			<0.001 *	<0.001 *		<0.001 *
Race/Ethnicity						
Non-Hispanic white	1824 (79.1)	1460 (81.5)	364 (69.2)	820 (77.1)	598 (85.6)	116 (66.6)
Non-Hispanic black	823 (8.1)	571 (7.2)	252 (11.7)	401 (8.2)	134 (4.9)	105 (16.9)
Mexican American	409 (3.8)	259 (3.0)	150 (7.1)	220 (4.1)	78 (2.8)	41 (6.5)
Other Hispanic	409 (3.6)	306 (3.3)	103 (4.5)	213 (3.7)	67 (2.4)	32 (3.9)
Non-Hispanic Asian	245 (3.6)	183 (3.4)	62 (4.6)	177 (5.3)	38 (2.1)	12 (3.8)
Other race	70 (1.8)	49 (1.5)	21 (2.8)	33 (1.5)	18 (2.2)	8 (2.4)
*p*-value			<0.001 *	0.003 *	0.003 *	0.003 *
Education Levels						
<High school	949 (15.1)	634 (13.1)	315 (23.0)	444 (13.9)	184 (12.5)	113 (28.6)
High school graduate	894 (22.3)	662 (21.4)	232 (26.0)	447 (22.0)	192 (21.1)	80 (22.4)
Some college	1072 (31.6)	836 (32.5)	236 (28.0)	488 (28.6)	300 (33.0)	92 (39.7)
≥College graduate	865 (31.0)	696 (33.0)	169 (23.0)	485 (35.5)	257 (33.4)	29 (9.3)
*p*-value			<0.001 *	<0.001 *	<0.001 *	<0.001 *
Income Status (PIR)						
Low (<130%)	1151 (17.8)	807 (16.5)	344 (22.8)	530 (16.4)	239 (14.3)	136 (32.2)
Medium (130–350%)	1518 (39.0)	1114 (38.1)	404 (42.6)	748 (37.0)	378 (40.3)	124 (43.1)
High (≥350%)	1111 (43.2)	907 (45.4)	204 (34.6)	586 (46.5)	316 (45.4)	54 (24.7)
*p*-value			0.005 *	<0.001 *	<0.001 *	<0.001 *
Weight Status						
Underweight/Healthy	942 (25.2)	796 (28.6)	146 (11.7)	572 (30.4)	189 (22.4)	58 (23.0)
Overweight	1334 (35.6)	1052 (37.4)	282 (28.4)	721 (39.0)	283 (30.6)	109 (33.2)
Obese	1504 (39.2)	980 (34.0)	524 (59.9)	571 (30.6)	461 (47.0)	147 (43.9)
*p*-value			<0.001 *	<0.001 *		<0.001 *
Physical Activity Levels ^a^						
Low	1986 (47.2)	1401 (45.1)	585 (55.8)	891 (42.2)	504 (46.7)	188 (57.5)
Medium	438 (12.2)	339 (12.1)	99 (12.8)	231 (13.3)	108 (12.0)	33 (10.6)
High	1356 (40.6)	1088 (42.8)	268 (31.4)	742 (44.4)	321 (41.3)	93 (31.9)
*p*-value			0.003 *	<0.001 *		<0.001 *
Smoking Status						
Non-current smoker	3319 (89.8)	2479 (89.8)	840 (89.7)	1624 (89.7)	850 (92.0)	264 (85.2)
Current smoker	461 (10.2)	349 (10.2)	112 (10.3)	240 (10.3)	83 (8.0)	50 (14.8)
*p*-value			0.968		0.003 *	

Abbreviations: NHANES National Health and Nutrition Examination Survey; DM diabetes mellitus; AR arthritis; OA osteoarthritis; RA rheumatoid arthritis; PIR poverty income ratio. Numbers presented are unweighted counts and weighted percentages based on the NHANES complex survey design. *P*-values for without vs. with DM (in the with DM column) were obtained by Rao-Scott adjusted chi-square tests. *P*-values for without AR vs. with OA (in the without AR column), with OA vs. with RA (in the with OA column), and without AR vs. with RA (in the with RA column) were obtained by Bonferroni adjusted post-hoc pairwise chi-square tests, using Monte Carlo simulations based on the weighted percentages. Analysis by AR status included those with other AR: characteristics of those with other AR are not shown in tables. ^a^ Low = <150, medium = 150–<300, and high = ≥300 metabolic equivalents minutes per week. *—indicates a statistically significant difference with a *p*-value of less than 0.01.

**Table 3:** Dietary characteristics of younger US adults by self-reported diabetes mellitus status and arthritis status: NHANES 2011–2016.

	All Younger Adults *n* = 7988	Without DM *n* = 7361 (93.5%)	With DM *n* = 627 (6.5%)	Without AR *n* = 6743 (83.6%)	With OA *n* = 496 (7.0%)	With RA *n* = 256 (2.8%)
Total Score						
Q1 (0–42.2)	1795 (22.4)	1651 (22.2)	144 (24.6)	1472 (22.1)	126 (24.3)	71 (22.1)
Q2 (42.3–49.9)	1726 (21.2)	1583 (21.1)	143 (22.2)	1439 (20.7)	111 (23.7)	67 (28.3)
Q3 (50.0–57.4)	1536 (19.3)	1413 (19.2)	123 (20.6)	1299 (19.2)	95 (19.9)	44 (17.0)
Q4 (57.5–66.2)	1523 (19.3)	1401 (19.3)	122 (18.9)	1308 (19.8)	77 (14.1)	47 (20.6)
Q5 (66.3–100)	1408 (17.9)	1313 (18.2)	95 (13.7)	1225 (18.3)	87 (18.1)	27 (11.9)
*p*-value			0.489			
Total Fruits						
Q1 (0–0.8)	2830 (36.2)	2601 (36.1)	229 (38.5)	2340 (35.7)	191 (41.2)	100 (30.1)
Q2 (0.9–3.4)	2485 (32.6)	2276 (32.4)	209 (35.1)	2111 (32.5)	155 (34.2)	68 (29.7)
Q3 (3.5–5)	2673 (31.2)	2484 (31.5)	189 (26.3)	2292 (31.8)	150 (24.7)	88 (40.2)
*p*-value			0.250			
Whole Fruits						
Q1 (0–0.3)	3012 (37.3)	2771 (36.9)	241 (43.5)	2505 (37.1)	197 (41.4)	111 (35.9)
Q2 (0.4–4.8)	2440 (32.0)	2247 (32.1)	193 (31.4)	2062 (31.8)	151 (32.2)	66 (26.0)
Q3 (4.9–5)	2536 (30.7)	2343 (31.1)	193 (25.1)	2176 (31.1)	148 (26.4)	79 (38.1)
*p*-value			0.063			
Total Vegetables						
Q1 (0–2.4)	2870 (35.1)	2677 (35.6)	193 (28.4)	2401 (35.2)	196 (35.3)	101 (43.5)
Q2 (2.5–4.2)	2639 (33.5)	2422 (33.2)	217 (36.9)	2234 (33.7)	145 (30.2)	90 (32.3)
Q3 (4.3–5)	2479 (31.4)	2262 (31.2)	217 (34.7)	2108 (31.1)	155 (34.5)	65 (24.2)
*p*-value			0.140			
Greens and Beans						
Q1 (0–1.3)	3941 (49.4)	3614 (49.1)	327 (53.6)	3262 (48.4)	277 (56.5)	143 (61.9)
Q2 (1.4–5)	4047 (50.6)	3747 (50.9)	300 (46.4)	3481 (51.6)	219 (43.5)	113 (38.1)
*p*-value			0.205			0.009 *
Whole Grains						
Q1 (0–0.5)	3091 (35.4)	2862 (35.5)	229 (34.1)	2602 (35.2)	185 (37.5)	110 (32.6)
Q2 (0.6–3.6)	2585 (32.9)	2380 (33.0)	205 (32.0)	2193 (33.5)	157 (30.3)	82 (34.7)
Q3 (3.7–5)	2312 (31.6)	2119 (31.5)	193 (33.9)	1948 (31.4)	154 (32.3)	64 (32.7)
*p*-value			0.716			
Dairy						
Q1 (0–3.6)	2990 (33.0)	2723 (33.0)	267 (32.9)	2493 (32.8)	178 (30.3)	107 (31.7)
Q2 (3.7–6.7)	2537 (32.5)	2339 (32.2)	198 (36.3)	2164 (32.4)	161 (36.3)	77 (33.2)
Q3 (6.8–10)	2461 (34.6)	2299 (34.8)	162 (30.8)	2086 (34.7)	157 (33.4)	72 (35.1)
*p*-value			0.321			
Total Protein Foods						
Q1 (0–4.9)	2574 (33.9)	2411 (34.3)	163 (29.1)	2161 (33.8)	160 (32.0)	98 (46.4)
Q2 (5)	5414 (66.1)	4950 (65.7)	464 (70.9)	4582 (66.2)	336 (68.0)	158 (53.6)
*p*-value			0.179			
Seafood and Plant Proteins						
Q1 (0–3.7)	4083 (51.5)	3756 (51.2)	327 (55.2)	3393 (50.9)	257 (48.5)	163 (67.9)
Q2 (3.8–5)	3905 (48.5)	3605 (48.8)	300 (44.8)	3350 (49.1)	239 (51.5)	93 (32.1)
*p*-value			0.286			<0.001 *
Fatty Acids						
Q1 (0–2.9)	2494 (33.6)	2329 (33.7)	165 (31.1)	2059 (33.0)	179 (35.1)	94 (43.2)
Q2 (3.0–6.5)	2594 (33.1)	2380 (32.9)	214 (36.1)	2221 (33.6)	149 (32.5)	73 (25.3)
Q3 (6.6–10)	2900 (33.3)	2652 (33.4)	248 (32.8)	2463 (33.4)	168 (32.4)	89 (31.5)
*p*-value			0.520		<0.001*	
Refined Grains						
Q1 (0–4.7)	2914 (35.2)	2671 (35.0)	243 (37.6)	2528 (35.4)	159 (35.4)	79 (30.8)
Q2 (4.8–8.6)	2646 (32.5)	2440 (32.6)	206 (31.8)	2218 (32.9)	165 (29.7)	99 (36.1)
Q3 (8.7–10)	2428 (32.3)	2250 (32.4)	178 (30.6)	1997 (31.7)	172 (34.9)	78 (33.1)
*p*-value			0.737			
Sodium						
Q1 (0–1.8)	2669 (33.5)	2382 (32.8)	287 (43.9)	2268 (34.1)	156 (32.7)	82 (23.9)
Q2 (1.9–5.3)	2665 (33.5)	2468 (33.4)	197 (35.2)	2240 (32.9)	177 (36.1)	90 (36.6)
Q3 (5.4–10)	2654 (33.0)	2511 (33.8)	143 (20.9)	2235 (33.0)	163 (31.2)	84 (39.5)
*p*-value			<0.001 *			
Added Sugars						
Q1 (0–5.9)	2892 (35.1)	2721 (35.7)	171 (25.2)	2362 (34.2)	206 (36.9)	124 (43.9)
Q2 (6.0–9.2)	2540 (31.3)	2362 (31.5)	178 (29.4)	2179 (31.4)	143 (32.5)	73 (34.8)
Q3 (9.3–10)	2556 (33.6)	2278 (32.8)	278 (45.3)	2202 (34.4)	147 (30.6)	59 (21.2)
*p*-value			<0.001 *			0.006 *
Saturated Fats						
Q1 (0–4.5)	2397 (32.3)	2165 (31.5)	232 (43.5)	1967 (31.4)	179 (37.6)	75 (29.6)
Q2 (4.6–7.8)	2546 (33.4)	2352 (33.6)	194 (30.4)	2157 (33.9)	148 (32.8)	95 (38.0)
Q3 (7.9–10)	3045 (34.3)	2844 (34.9)	201 (26.0)	2619 (34.7)	169 (29.6)	86 (32.4)
*p*-value			<0.001 *			

Abbreviations: NHANES National Health and Nutrition Examination Survey; DM diabetes mellitus; AR arthritis; OA osteoarthritis; RA rheumatoid arthritis. Numbers presented are unweighted counts and weighted percentages based on the NHANES complex survey design. *P*-values for without vs. with DM (in the with DM column) were obtained by Rao-Scott adjusted chi-square tests. *P*-values for without AR vs. with OA (in the without AR column), with OA vs. with RA (in the with OA column), and without AR vs. with RA (in the with RA column) were obtained by Bonferroni adjusted post-hoc pairwise chi-square tests, using Monte Carlo simulations based on the weighted percentages. Analysis by AR status included those with other AR: characteristics of those with other AR are not shown in Tables. *—indicates a statistically significant difference with a *p*-value of less than 0.01.

**Table 4:** Dietary characteristics of older US adults by self-reported diabetes mellitus status and arthritis status: NHANES 2011–2016.

	All Older Adults *n* = 3780	Without DM *n* = 2828 (80.0%)	With DM *n* = 952 (20.0%)	Without AR *n* = 1864 (46.6%)	With OA *n* = 933 (31.5%)	With RA *n* = 314 (5.8%)
Total Score						
Q1 (0–42.2)	534 (13.3)	386 (13.0)	148 (14.4)	251 (11.3)	111 (11.6)	62 (21.6)
Q2 (42.3–49.9)	634 (16.8)	480 (16.7)	154 (17.2)	315 (16.3)	142 (14.8)	58 (15.8)
Q3 (50.0–57.4)	792 (21.9)	586 (22.2)	206 (20.8)	373 (21.5)	197 (21.0)	76 (25.5)
Q4 (57.5–66.2)	842 (22.0)	623 (21.6)	219 (23.8)	408 (23.5)	222 (23.2)	63 (15.8)
Q5 (66.3–100)	978 (26.0)	753 (26.5)	225 (23.9)	517 (27.5)	261 (29.5)	55 (21.2)
*p*-value			0.757		<0.001 *	<0.001 *
Total Fruits						
Q1 (0–0.8)	907 (23.8)	628 (22.7)	279 (28.3)	448 (23.7)	192 (19.7)	81 (26.7)
Q2 (0.9–3.4)	1177 (34.1)	904 (34.2)	273 (33.5)	570 (31.4)	298 (37.2)	106 (37.9)
Q3 (3.5–5)	1696 (42.1)	1296 (43.1)	400 (38.2)	846 (44.9)	443 (43.1)	127 (35.3)
*p*-value			0.089	<0.001 *	<0.001 *	<0.001 *
Whole Fruits						
Q1 (0–0.3)	904 (20.9)	643 (19.6)	261 (26.3)	451 (20.5)	181 (16.8)	94 (30.0)
Q2 (0.4–4.8)	1231 (35.7)	937 (37.0)	294 (30.6)	590 (33.4)	306 (38.3)	105 (37.8)
Q3 (4.9–5)	1645 (43.3)	1248 (43.4)	397 (43.1)	823 (46.1)	446 (44.8)	115 (32.2)
*p*-value			0.053	0.002 *	0.002 *	
Total Vegetables						
Q1 (0–2.4)	1104 (27.3)	833 (27.6)	271 (26.3)	531 (28.0)	246 (25.4)	105 (29.1)
Q2 (2.5–4.2)	1164 (31.5)	877 (31.2)	287 (32.5)	561 (29.2)	306 (34.4)	96 (34.5)
Q3 (4.3–5)	1512 (41.2)	1118 (41.2)	394 (41.2)	772 (42.8)	381 (40.3)	113 (36.4)
*p*-value			0.870			
Greens and Beans						
Q1 (0–1.3)	1867 (51.5)	1385 (50.7)	482 (54.4)	873 (49.2)	465 (48.9)	182 (61.8)
Q2 (1.4–5)	1913 (48.5)	1443 (49.3)	470 (45.6)	991 (50.8)	468 (51.1)	132 (38.2)
*p*-value			0.258			0.009 *
Whole Grains						
Q1 (0–0.5)	1088 (26.1)	817 (25.5)	271 (28.7)	533 (25.0)	241 (24.5)	105 (29.3)
Q2 (0.6–3.6)	1171 (33.2)	872 (33.6)	299 (31.7)	569 (33.0)	294 (31.3)	98 (35.9)
Q3 (3.7–5)	1521 (40.7)	1139 (40.9)	382 (39.6)	762 (42.0)	398 (44.2)	111 (34.8)
*p*-value			0.470			
Dairy						
Q1 (0–3.6)	1470 (33.2)	1079 (32.4)	391 (36.1)	731 (30.9)	339 (34.4)	129 (32.1)
Q2 (3.7–6.7)	1188 (34.3)	898 (34.8)	290 (32.0)	585 (35.3)	310 (34.0)	95 (29.9)
Q3 (6.8–10)	1122 (32.6)	851 (32.8)	271 (31.9)	548 (33.8)	284 (31.6)	90 (37.9)
*p*-value			0.391			
Total Protein Foods						
Q1 (0–4.9)	1142 (33.6)	920 (36.0)	222 (23.9)	542 (33.7)	297 (31.5)	102 (32.3)
Q2 (5)	2638 (66.4)	1908 (64.0)	730 (76.1)	1322 (66.3)	636 (68.5)	212 (67.7)
*p*-value			<0.001 *			
Seafood and Plant Proteins						
Q1 (0–3.7)	1706 (45.9)	1305 (47.2)	401 (40.8)	800 (43.1)	417 (44.2)	169 (54.6)
Q2 (3.8–5)	2074 (54.1)	1523 (52.8)	551 (59.2)	1064 (56.9)	516 (55.8)	145 (45.4)
*p*-value			0.028			
Fatty Acids						
Q1 (0–2.9)	1126 (31.4)	873 (32.0)	253 (29.2)	562 (32.1)	275 (28.2)	92 (31.6)
Q2 (3.0–6.5)	1198 (32.9)	875 (32.3)	323 (35.1)	569 (32.5)	305 (34.9)	114 (35.6)
Q3 (6.6–10)	1456 (35.7)	1080 (35.7)	376 (35.8)	733 (35.4)	353 (36.9)	108 (32.8)
*p*-value			0.657			
Refined Grains						
Q1 (0–4.7)	1161 (27.1)	823 (26.5)	338 (29.2)	582 (27.3)	258 (24.1)	99 (30.6)
Q2 (4.8–8.6)	1213 (34.3)	901 (33.3)	312 (38.3)	583 (33.3)	308 (36.1)	115 (35.8)
Q3 (8.7–10)	1406 (38.7)	1104 (40.2)	302 (32.5)	699 (39.5)	367 (39.7)	100 (33.7)
*p*-value			0.073			
Sodium						
Q1 (0–1.8)	1254 (31.7)	845 (29.6)	409 (40.0)	614 (30.0)	286 (29.4)	110 (31.7)
Q2 (1.9–5.3)	1199 (31.7)	899 (31.2)	300 (33.5)	609 (34.1)	288 (29.3)	90 (29.8)
Q3 (5.4–10)	1327 (36.6)	1084 (39.2)	243 (26.5)	641 (35.9)	359 (41.4)	114 (38.6)
*p*-value			<0.001 *	<0.001 *		
Added Sugars						
Q1 (0–5.9)	1024 (26.9)	841 (29.1)	183 (18.3)	490 (25.5)	254 (26.6)	96 (32.4)
Q2 (6.0–9.2)	1349 (38.2)	1048 (38.9)	301 (35.3)	646 (36.3)	349 (41.3)	104 (33.3)
Q3 (9.3–10)	1407 (34.9)	939 (32.0)	468 (46.4)	728 (38.3)	330 (32.1)	114 (34.3)
*p*-value			<0.001 *	<0.001 *		
Saturated Fats						
Q1 (0–4.5)	1190 (35.0)	892 (34.8)	298 (35.6)	574 (35.9)	305 (31.1)	100 (35.7)
Q2 (4.6–7.8)	1193 (32.1)	881 (31.7)	312 (33.7)	538 (28.9)	308 (36.3)	115 (37.3)
Q3 (7.9–10)	1397 (32.9)	1055 (33.5)	342 (30.7)	752 (35.2)	320 (32.7)	99 (27.1)
*p*-value			0.669	<0.001 *		

Abbreviations: NHANES National Health and Nutrition Examination Survey; DM diabetes mellitus; AR arthritis; OA osteoarthritis; RA rheumatoid arthritis. Numbers presented are unweighted counts and weighted percentages based on the NHANES complex survey design. *P*-values for without vs. with DM (in the with DM column) were obtained by Rao-Scott adjusted chi-square tests. *P*-values for without AR vs. with OA (in the without AR column), with OA vs. with RA (in the with OA column), and without AR vs. with RA (in the with RA column) were obtained by Bonferroni adjusted post-hoc pairwise chi-square tests, using Monte Carlo simulations based on the weighted percentages. Analysis by AR status included those with other AR: characteristics of those with other AR are not shown in Tables. *—indicates a statistically significant difference with a *p*-value of less than 0.01.

**Table 5:** Odds ratios and 99% confidence intervals of self-reported diabetes mellitus, osteoarthritis, and rheumatoid arthritis among younger and older US adults from the initial Models with the Healthy Eating Index-2015 total and component scores: NHANES 2011–2016.

	Self-Reported DM		Self-Reported OA		Self-Reported RA	
	Younger Adults	Older Adults	Younger Adults	Older Adults	Younger Adults	Older Adults
Model with Total Score						
Q1	1.00	1.00	1.00	1.00	1.00	1.00
Q2	1.01 (0.56–1.80)	1.03 (0.57–1.86)	1.16 (0.70–1.94)	0.79 (0.38–1.64)	1.49 (0.79–2.83)	0.52 (0.25–1.10)
Q3	1.15 (0.63–2.12)	0.97 (0.45–2.09)	1.14 (0.58–2.23)	0.87 (0.50–1.52)	1.13 (0.57–2.22)	0.72 (0.30–1.75)
Q4	1.12 (0.59–2.12)	1.36 (0.71–2.59)	0.83 (0.45–1.53)	0.90 (0.51–1.58)	1.55 (0.73–3.33)	0.35 (0.16–0.76) *
Q5	1.08 (0.50–2.34)	1.29 (0.65–2.55)	1.29 (0.66–2.53)	1.01 (0.54–1.88)	1.08 (0.45–2.61)	0.58 (0.24–1.41)
Model with Components						
Total Fruits						
Q1	1.00	1.00	1.00	1.00	1.00	1.00
Q2	1.31 (0.74–2.32)	0.87 (0.60–1.28)	0.98 (0.58–1.64)	0.97 (0.86–1.08)	1.35 (0.60–3.04)	1.85 (0.94–3.65)
Q3	1.17 (0.60–2.28)	0.70 (0.34–1.42)	0.73 (0.37–1.44)	0.97 (0.85–1.12)	1.74 (0.49–6.18)	1.68 (0.71–3.99)
Whole Fruits						
Q1	1.00	1.00	1.00	1.00	1.00	1.00
Q2	0.81 (0.48–1.34)	0.71 (0.37–1.38)	1.17 (0.77–1.76)	1.02 (0.89–1.18)	1.00 (0.44–2.27)	0.68 (0.37–1.28)
Q3	0.68 (0.33–1.42)	1.24 (0.46–3.34)	1.23 (0.62–2.46)	1.04 (0.85–1.28)	1.42 (0.49–4.13)	0.43 (0.21–0.85) *
Total Vegetables						
Q1	1.00	1.00	1.00	1.00	1.00	1.00
Q2	1.37 (0.80–2.34)	1.18 (0.75–1.83)	1.03 (0.75–1.40)	1.00 (0.88–1.14)	0.99 (0.60–1.65)	1.31 (0.61–2.79)
Q3	1.36 (0.90–2.07)	1.03 (0.64–1.68)	1.52 (1.00–2.31)	0.99 (0.86–1.15)	0.84 (0.41–1.73)	1.24 (0.53–2.92)
Greens Beans						
Q1	1.00	1.00	1.00	1.00	1.00	1.00
Q2	0.88 (0.59–1.30)	0.78 (0.53–1.15)	0.70 (0.47–1.04)	1.00 (0.91–1.10)	0.87 (0.52–1.45)	0.74 (0.41–1.31)
Whole Grains						
Q1	1.00	1.00	1.00	1.00	1.00	1.00
Q2	1.20 (0.83–1.74)	0.90 (0.59–1.37)	0.88 (0.56–1.40)	0.99 (0.88–1.12)	1.27 (0.62–2.61)	0.99 (0.62–1.59)
Q3	1.52 (1.01–2.29) *	0.94 (0.57–1.54)	1.09 (0.63–1.88)	0.98 (0.87–1.12)	1.40 (0.61–3.22)	0.83 (0.41–1.67)
Dairy						
Q1	1.00	1.00	1.00	1.00	1.00	1.00
Q2	1.15 (0.72–1.84)	0.98 (0.64–1.49)	1.18 (0.77–1.82)	1.01 (0.89–1.13)	0.98 (0.49–1.96)	1.07 (0.61–1.88)
Q3	0.89 (0.50–1.61)	1.02 (0.63–1.65)	1.02 (0.62–1.68)	1.01 (0.89–1.15)	0.85 (0.43–1.69)	1.46 (0.69–3.05)
Total Protein						
Q1	1.00	1.00	1.00	1.00	1.00	1.00
Q2	0.90 (0.55–1.47)	1.28 (0.90–1.83)	1.21 (0.78–1.87)	1.00 (0.90–1.12)	0.82 (0.47–1.42)	1.25 (0.61–2.56)
Seafood/Plant						
Q1	1.00	1.00	1.00	1.00	1.00	1.00
Q2	0.98 (0.67–1.44)	1.49 (1.02–2.16)	1.45 (0.96–2.20)	1.00 (0.90–1.10)	0.76 (0.43–1.34)	0.73 (0.40–1.32)
Fatty Acids						
Q1	1.00	1.00	1.00	1.00	1.00	1.00
Q2	1.14 (0.71–1.84)	1.04 (0.58–1.85)	0.84 (0.51–1.39)	1.00 (0.90–1.12)	0.54 (0.29–0.99)	1.34 (0.49–3.61)
Q3	1.29 (0.69–2.40)	0.90 (0.49–1.65)	0.89 (0.62–1.29)	1.02 (0.90–1.15)	0.75 (0.37–1.56)	1.34 (0.55–3.30)
Refined Grains						
Q1	1.00	1.00	1.00	1.00	1.00	1.00
Q2	1.01 (0.59–1.73)	1.38 (0.81–2.33)	0.74 (0.49–1.10)	1.00 (0.91–1.11)	1.21 (0.77–1.91)	1.08 (0.60–1.95)
Q3	1.13 (0.68–1.90)	1.01 (0.61–1.67)	0.92 (0.62–1.36)	0.98 (0.87–1.11)	1.13 (0.62–2.03)	0.89 (0.44–1.79)
Sodium						
Q1	1.00	1.00	1.00	1.00	1.00	1.00
Q2	0.88 (0.58–1.35)	0.95 (0.62–1.45)	1.24 (0.85–1.82)	1.01 (0.91–1.13)	1.29 (0.72–2.31)	1.03 (0.52–2.05)
Q3	0.60 (0.33–1.08)	0.76 (0.44–1.30)	1.11 (0.73–1.68)	1.01 (0.88–1.16)	1.10 (0.56–2.16)	1.59 (0.93–2.74)
Added Sugars						
Q1	1.00	1.00	1.00	1.00	1.00	1.00
Q2	1.23 (0.83–1.83)	1.57 (0.96–2.57)	1.04 (0.70–1.56)	0.99 (0.88–1.10)	1.11 (0.54–2.29)	0.91 (0.51–1.63)
Q3	2.08 (1.22–3.55) *	2.34 (1.50–3.65) *	0.80 (0.48–1.34)	0.96 (0.84–1.10)	0.66 (0.37–1.19)	0.93 (0.46–1.87)
Saturated Fats						
Q1	1.00	1.00	1.00	1.00	1.00	1.00
Q2	0.64 (0.38–1.06)	1.00 (0.61–1.64)	0.84 (0.52–1.35)	0.98 (0.86–1.12)	1.37 (0.76–2.49)	1.29 (0.65–2.58)
Q3	0.53 (0.34–0.82) *	0.86 (0.47–1.60)	0.87 (0.52–1.45)	0.99 (0.86–1.15)	1.15 (0.62–2.13)	0.81 (0.40–1.67)

Abbreviations: NHANES National Health and Nutrition Examination Survey; DM diabetes mellitus; OA osteoarthritis; RA rheumatoid arthritis. Each logistic regression model included the Healthy Eating Index-2015 total score or 13 component scores and covariates (self-reported disease status, sex, race, education level, income status, weight status, physical activity level, and smoking status). *—indicates a statistically significant difference with a *p*-value of less than 0.01.

**Table 6:** Adjusted odds ratios and 99% confidence intervals for self-reported diabetes mellitus, osteoarthritis, and rheumatoid arthritis among younger and older US adults from the final Models with the Healthy Eating Index 2015 component scores: NHANES 2011–2016.

	Self-Reported DM		Self-Reported OA		Self-Reported RA	
	Younger Adults	Older Adults	Younger Adults	Older Adults	Younger Adults	Older Adults
Sodium						
Q1	1.00					
Q2	0.86 (0.56–1.31)					
Q3	0.56 (0.33–0.95) *					
Added Sugars						
Q1	1.00	1.00				
Q2	1.38 (0.89–2.13)	1.66 (0.99–2.79)				
Q3	2.31 (1.33–4.02) *	2.54 (1.62–3.97) *				
Saturated Fats						
Q1				1.00		
Q2				1.54 (1.08–2.19) *		
Q3				1.24 (0.82–1.86)		
Seafood and Plant Proteins						
Q1					1.00	
Q2					0.58 (0.35–0.92) *	
Arthritis Status						
Without arthritis	1.00					
With OA	2.50 (1.44–4.35) *					
With RA	3.51 (1.72–7.15) *					
With Other arthritis	2.57 (1.44–4.35) *					
Diabetes Mellitus Status						
Without DM			1.00		1.00	
With DM			2.76 (1.48–5.14) *		3.86 (2.17–6.85) *	
Gender						
Male		1.00	1.00	1.00	1.00	
Female		0.56 (0.41–0.77) *	1.88 (1.22–2.90) *	2.49 (1.77–3.5) *	1.89 (1.17–3.05) *	
Race/Ethnicity						
Non-Hispanic White		1.00	1.00	1.00	1.00	1.00
Non-Hispanic Black		1.77 (1.31–2.39) *	0.55 (0.36–0.84) *	0.45 (0.28–0.72) *	0.88 (0.49–1.57)	1.88 (1.20–2.95) *
Mexican American		1.78 (1.25–2.54) *	0.24 (0.13–0.44) *	0.56 (0.37–0.84) *	0.42 (0.20–0.91) *	1.12 (0.66–1.89)
Other Hispanic		1.26 (0.90–1.78)	0.37 (0.20–0.70) *	0.55 (0.25–1.19)	0.83 (0.37–1.87)	0.80 (0.40–1.59)
Non-Hispanic Asian		2.28 (1.19–4.34) *	0.25 (0.12–0.55) *	0.44 (0.25–0.77) *	0.16 (0.03–0.81) *	0.92 (0.35–2.73)
Other Race		2.36 (0.98–5.70)	0.84 (0.44–1.58)	1.10 (0.41–3.00)	0.63 (0.20–1.94)	1.47 (0.41–5.48)
Education Levels						
<High school	1.00	1.00			1.00	1.00
High school graduate	0.73 (0.39–1.36)	0.79 (0.54–1.17)			0.64 (0.29–1.39)	0.51 (0.27–0.97)
Some college	0.82 (0.48–1.38)	0.53 (0.32–0.87) *			0.67 (0.32–1.38)	0.71 (0.37–1.36)
≥College graduate	0.43 (0.21–0.88) *	0.42 (0.27–0.64) *			0.29 (0.12–0.73) *	0.14 (0.06–0.30) *
Weight Status						
Underweight/Healthy	1.00	1.00	1.00	1.00		
Overweight	1.63 (0.91–2.94)	1.83 (1.19–2.80) *	1.23 (0.76–2.00)	1.21 (0.70–2.10)		
Obese	5.06 (2.65–9.67) *	4.44 (2.98–6.61) *	2.00 (1.21–3.31) *	2.35 (1.60–3.45) *		
Physical Activity Levels ^a^						
Low	1.00		1.00			
Medium	0.55 (0.33–0.96) *		0.51 (0.21–1.25)			
High	0.71 (0.49–1.01)		0.58 (0.39–0.85) *			
Smoking Status						
Non-current smoker			1.00			
Current smoker			1.89 (1.28–2.78) *			

Abbreviations: NHANES National Health and Nutrition Examination Survey; DM diabetes mellitus; OA osteoarthritis; RA rheumatoid arthritis; AR arthritis. ^a^ Low = <150, medium = 150–<300, and high = ≥300 metabolic equivalents minutes per week. *—indicates a statistically significant difference with a *p*-value of less than 0.01.

## Data Availability

The data used in this study are openly available in the website of the National Health and Nutrition Examination Survey: NHANES Questionnaires, Datasets, and Related Documentation: https://wwwn.cdc.gov/nchs/nhanes/Default.aspx.
